# A Quantitative Examination of Extreme Facial Pain Expression in Neonates: The Primal Face of Pain across Time

**DOI:** 10.1155/2012/251625

**Published:** 2012-05-07

**Authors:** Martin Schiavenato, Carl L. von Baeyer

**Affiliations:** ^1^School of Nursing and Health Studies, University of Miami, 5030 Brunson Drive, Coral Gables, FL 33146, USA; ^2^Departments of Psychology and Pediatrics, University of Saskatchewan, Saskatoon, SK S7N 5A5, Canada

## Abstract

Many pain assessment tools for preschool and school-aged children are based on facial expressions of pain. Despite broad use, their metrics are not rooted in the anatomic display of the facial pain expression. We aim to describe quantitatively the patterns of initiation and maintenance of the infant pain expression across an expressive cycle. We evaluated the trajectory of the pain expression of three newborns with the most intense facial display among 63 infants receiving a painful stimulus. A modified “point-pair” system was used to measure movement in key areas across the face by analyzing still pictures from video recording the procedure. Point-pairs were combined into “upper face” and “lower face” variables; duration and intensity of expression were standardized. Intensity and duration of expression varied among infants. Upper and lower face movement rose and overlapped in intensity about 30% into the expression. The expression reached plateau without major change for the duration of the expressive cycle. We conclude that there appears to be a shared pattern in the dynamic trajectory of the pain display among infants expressing extreme intensity. We speculate that these patterns are important in the communication of pain, and their incorporation in facial pain scales may improve current metrics.

## 1. Introduction

Facial expression is a cornerstone of both observational and self-report measurement of pediatric pain [1–4].  Table 1 lists commonly published pain scales that incorporate facial expression in their usage; the list, though not exhaustive, clearly illustrates the widely accepted use of facial expression as the currency in the communication and clinical evaluation of pain in children across the age spectrum, from neonates to school-aged. We have proposed that the *primal face of pain *(PFP), that common, inborn expression universally present in humans and prototypically displayed in neonates, is the foundation for our recognition of pain [5]. If this is the case, then the PFP is significant in understanding and possibly improving our current methodology of pain measurement in children. For example, it is important to assess the role of the PFP in the context of faces pain scales (FPSs) since the nature of these tools is to measure pain by graphic representation of facial displays showing progressive levels of distress. These self-report pain assessment tools are generally used with preschool and school-aged children (3–12 years old) [6, 7]. However, despite the broad use of FPSs, little empirical work has been done to assess the progression and timing of the behavioral pain display and its relation to current scaling schemes. Rather, current popular FPSs have been developed by relying on children's drawings of facial pain expressions, on others' judgment of the pain display (i.e., subjective rating of a graphic or photo), on artists' conception of the pain display, and on arbitrary intervals suited to facilitate counting rather than illustrate the trajectory or intensification of the display [8–11]. This approach is disconcerting because subtle graphic variations in FPSs are meaningful and impact the pain score [12]. Moreover, the ability to use FPSs is developmentally incremental [6, 13, 14]. Younger children in particular may benefit from a clearer or more accurate depiction of the pain display. Ultimately, effective clinical pain assessment hinges on the availability of valid tools, and their proper bedside application [15].

This study focuses on 3 selected infants showing extreme facial expression intensity to a common pain stimulus. Our goal is to examine the dynamics of their expression (i.e., the PFP), describe its progression and timing, and search for patterns, if any, along the pain display. If pain facial expression is universal with common features across time, then these patterns may hold important cues about the representation of pain intensity in FPSs and hold potential for their improvement by clarifying meaning and facilitating communication with younger children. A clearer understanding of the way humans express and subsequently recognize pain may advance assessment of pain at the bedside. 

## 2. Materials and Methods

### 2.1. Study Design and Sample

We previously reported on an evaluation of 63 term neonates of both sexes and various racial/ethnic backgrounds using a computerized “point-pair” method to measure facial movement in response to a painful stimulus [17]. Parental consent was obtained and the protocol was reviewed and approved by the ethics board. Briefly, these were healthy infants whose faces were video-recorded while receiving a heel-stick by the nursing staff for routine blood-draw diagnostic purposes. The point-pair method was used to assess the total intensity in facial expression across key anatomical areas of the face. Intensity was measured by number of pixels changed in facial movement between pairs of points from a *baseline* picture derived from the video (immediately before heel-stick) to a picture or video frame showing peak pain expression. Measurements were expressed as percent of facial width to preclude issues of individual differences in face anthropometrics and image size. In this analysis, we applied a similar methodology to that same data set and explored facial movement, but rather than measuring two video frames per infant, baseline and peak movement, we measured the change across each video frame, recorded at 11.6 frames per second, from baseline through to peak movement. We aimed to assess the progression of the PFP across time, thus the fullest or extreme display (i.e., *peak pain expression*) provided the best chance to do so descriptively. In other words, assuming that the PFP is the currency of facial pain expression, it is necessary to analyze infants displaying a full, peak, or prototypical facial expression of pain, thus characterizing *all* of the presumed range of pain expressions said to be portrayed by a common FPS (e.g., “0 no pain” to “10 worst pain.”). In order to select the three infants for the present study, we rank ordered the 63 infants on overall display intensity and identified the three infants whose display intensity was the highest (Infant A, Infant B, and Infant C) during this first expressive cycle as measured by the point-pair system [17]. See Table 2 for sample and case intensity measurements.

### 2.2. Expressive Cycle

**Figure 1:**
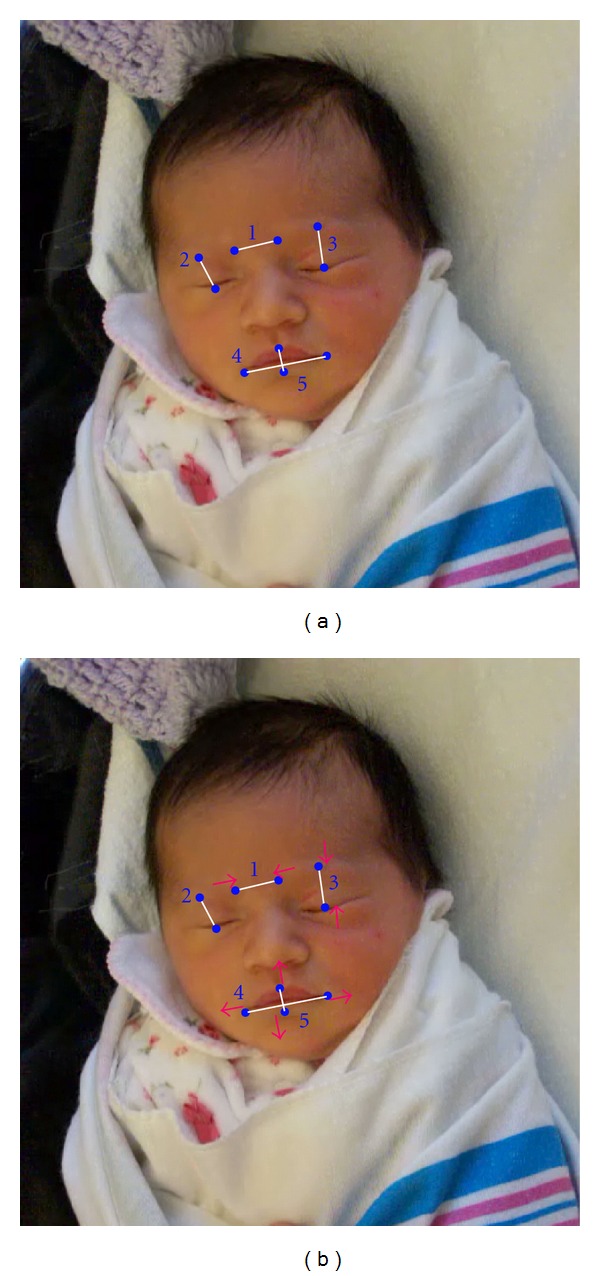
Point-pair scheme.

Observation reveals that the facial expression of pain in newborns following a heel-stick can be described as cyclic in nature and consisting of bursts of facial movement coinciding with the breathing cycle: exhalation accompanied by increased facial tension, followed by inhalation and a temporary relaxation of the movement, followed again by exhalation and facial tension, and so on. These cyclic patterns are more evident or characteristic of infants with higher display intensities. The *first expressive cycle* comprises facial action across time from baseline through to peak movement. We define *peak movement *as the point of maximal expression intensity during the first expressive cycle immediately following the pain stimulus as measured by our point-pair system. The specific physiologic movements of interest, long ago shown to be characteristic of neonatal pain expression [17, 18], are brow bulge (point-pair 1), eye squeeze (point-pairs 2 and 3), horizontal mouth movement (point-pair 4), and vertical mouth movement (point-pair 5); see “Point-pair scheme,” Figure 1.

### 2.3. Upper Face and Lower Face

**Figure 2:**
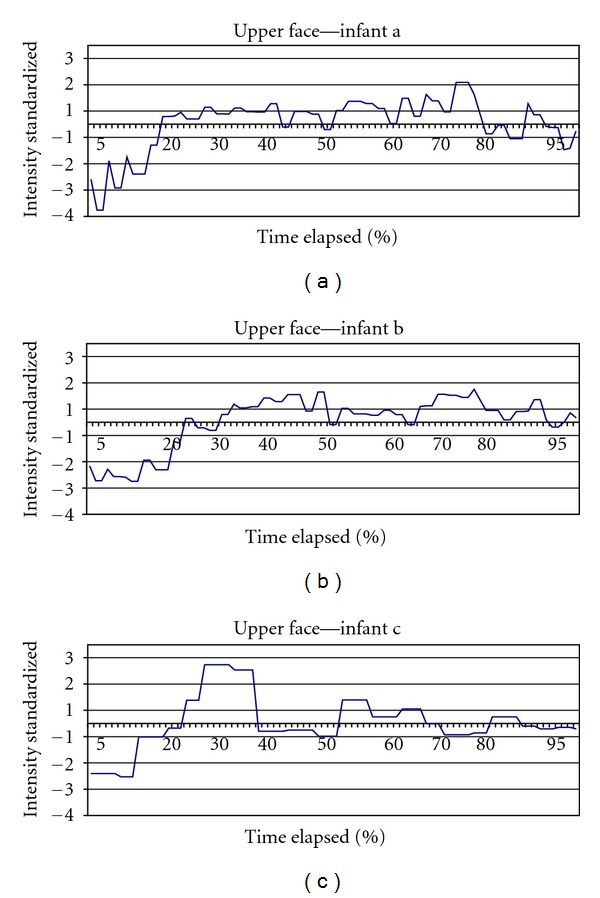
Upper face movement (sign reversed).

Pain expression occurs across two major groups of muscles, those controlling the upper face (eyes, eyebrows) and those controlling mouth movement [19]. Thus, we divided the first expressive cycle by upper and lower facial movement. We collapsed point-pairs 1, 2, and 3 and designated their total movement as the variable *upper face*. Similarly, point-pairs 4 and 5 were summed and designated as *lower face*. With upper face, point-pairs 1, 2, and 3 draw in towards each other (i.e., approximation of the brow and eyes); therefore, point distance decreases or is negative across the expression. The opposite occurs in the lower face, with point-pairs 4 and 5 moving away from each other (i.e., mouth is widening); there, point distance increases or is positive across the expression. To facilitate graphic comparisons between upper and lower face action, we switched the sign in upper facial action to match that of lower facial action (Figure 2). Since intensity of the expression varies across infants (i.e., no two infants display the exact physiologic movement intensity or strength of expression across the different facial actions), we standardized movement values using *z* scores. Likewise, duration of expressive cycle varied between infants; therefore, time was standardized as percent of the total duration of the selected expression, with baseline at 0 percent, and peak at 100 percent of the first expressive cycle. In conclusion, we observed the nature of the expression of those infants selected as displaying its fullest or most prototypical form, in order to search for common patterns that may be identified as universal and thus key in advancing clinical pain measurement.

## 3. Results

**Figure 3:**
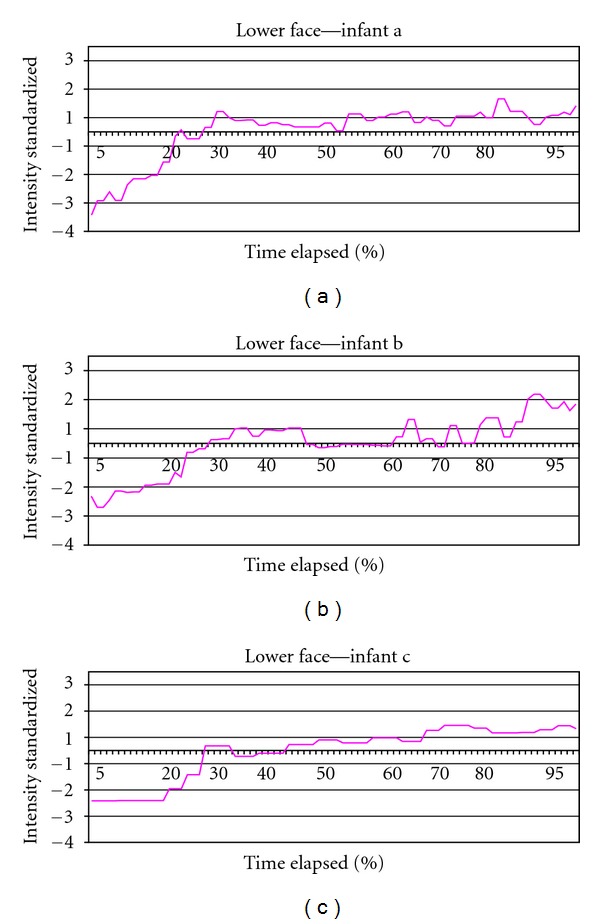
Lower face movement.

**Figure 4:**
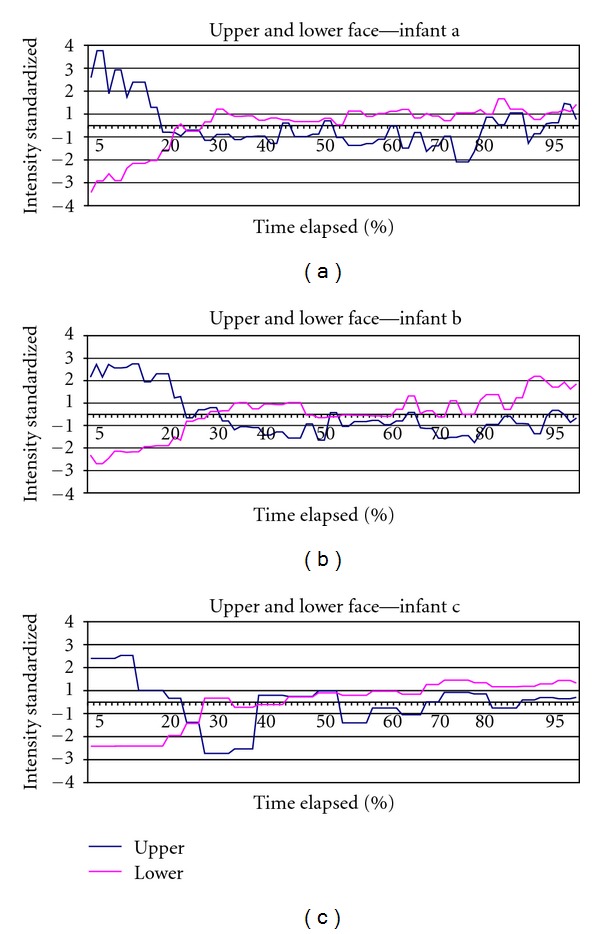
Upper and lower facial movement.

**Figure 5:**
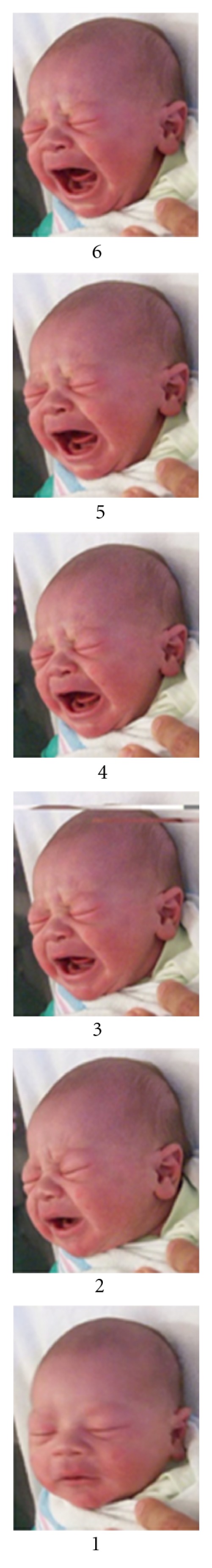
Expressive cycle at equal 20% intervals (infant A).

**Figure 6:**
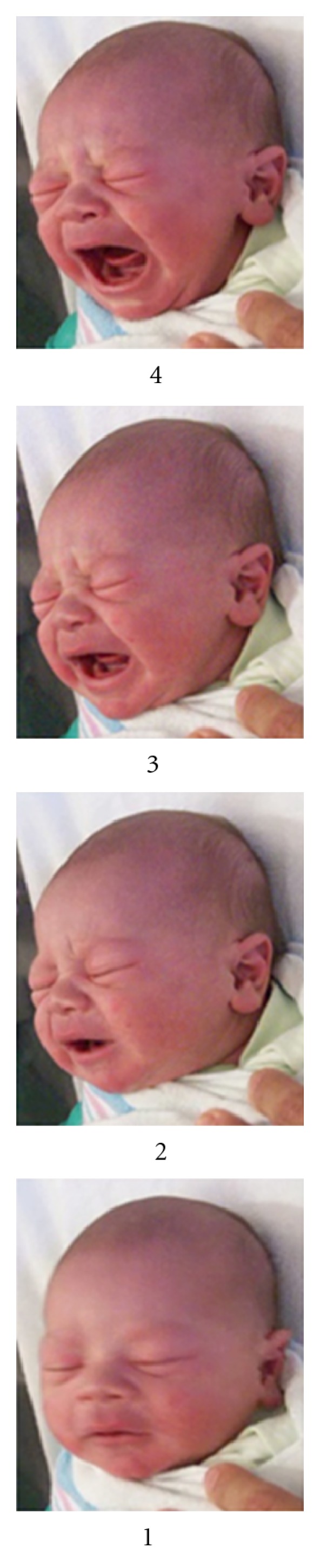
Expressive cycle at naturally occurring intervals (infant A).

Table 2 lists the characteristics of the 3 infants from the group of 63 displaying extreme facial intensity. The mean age of the infants was 42.3 hours. The mean duration for the first expressive cycle was 3.22 seconds and ranged among the 3 infants from 1.81 to 3.97 seconds in duration. Similarly, display intensity varied with a mean of 67.13 and a range of 55.46 to 80.69 among the 3. Full sample intensity figures are referenced at the bottom of the table. Number of picture frames analyzed per infant varied accordingly with expressive cycle duration. Figure 2 shows upper face standardized movement across time from beginning to end of expression for each of the three infants; sign is reversed to facilitate comparison with Figure 3. Figure 3 shows the same information for lower face action.  Figure 4 shows both upper and lower facial movement across expression for each infant. Figures 2 and 3 show comparable crescendo action early in the expression from the upper and lower face, respectively. As can be seen in Figure 4, which combines both upper and lower actions per infant, there is an overlap in peak action between the two areas at about 30% into the expression (Upper Face/Lower Face intersection). After this point, a plateau is sustained for the remaining of the expression without major intensity changes occurring.  Figure 5 illustrates the expressive cycle for Infant A from baseline to peak at equal intervals of 20% in a 6-interval scheme as the one currently in use in popular FPSs. Note the similarity in the pictures portraying levels 3, 4, and 5. These levels mark the 40%–80% interval, which are associated with the plateau of the expression and constitute at least half of the expressive cycle. In contrast, Figure 6 consists of baseline (level 1), immediately before Upper Face/Lower Face intersection (level 2), immediately after Upper Face/Lower Face intersection (level 3), and peak (level 4).

## 4. Discussion

Our previous analysis looked at intensity of pain expression in neonates across key areas of the face and found that although not identical, overall facial movement in reaction to a painful stimulus is quite similar in newborns regardless of their sex or race/ethnic background [17]. We termed this reaction the primal face of pain (PFP). Here we have looked at the intermediate steps along this expression, to describe how the PFP is initiated and developed within an expressive cycle. We chose to analyze infants displaying extreme intensity because they illustrate the full or maximum range of change in expression characteristic of current metric schemes (facial pain scales; FPSs), which generally display neutral, low, and high level intervals. Among just three infants, we found variety in the expression both by intensity (how much total facial tension) and by duration (time to reach peak expression). That is, there are unique individual timing and intensity subtleties in the facial pain display between infants; nevertheless, the key anatomical features of the PFP, its morphology, together with the dynamics of expression across time were the same among the three infants.

In more general terms, individual differences in emotional facial expression are greatly influenced by genetic mechanisms, especially in this population given the relative little social learning accessible to 2-day-old neonates [17, 20, 21]. Consequently, it could be said that the PFP is analogous to other inherited traits. For example, despite the fact that there is variation in the phenotype for the human nose, a nose is, nevertheless, easily recognizable both in appearance and function as such and not easily confused with, say, a human eye. If expressions are servants of communication, it would follow that the PFP would need to be unequivocally identifiable as a distress call, one that is intricately and importantly tied to species survival [22]. Thus, despite subtle variations, the expression itself must retain a significant level of commonality to ensure its function in communication. Specifically to our case, the facial expression of pain is shown here to have a common progression that delineates and explicitly heralds pain. This commonality is displayed in the shared morphology and temporal dynamics by these infants during the course of the expression. The PFP employs a common set of musculature [23], which serves a common evolutionary purpose, along, what appears to be a generally common pattern. These shared patterns or natural intervals denote the trajectory or intensification of expression from baseline to peak. Further, these natural intervals mark more defined changes in expression and do not occur at fixed times in relation to each other (e.g., at 20% increments or with 6 distinct intervals as commonly portrayed in FPSs); nevertheless, these patterns are consistent among the 3 infants.

The cases chosen for this detailed temporal analysis were those infants whose facial displays of pain in response to heel lance, measured by the point-pair method, were the most intense. There are many individual and technical influences on intensity of pain expression in this context, including the handling and the behavioral state of the infant before the procedure, the duration of the procedure, the possible lingering effects of obstetric medications and procedures, and potentially the sex and ethnicity of the infant. None of these other influences were controlled, raising the question of generalizability of the findings. However, while the intensity of pain expression in the present sample was not representative of all infants, we consider it unlikely that the sequence of movements identified here would have been influenced by such external factors.

It is reasonable to assume that the PFP is vital to the identification of pain or generalized distress in humans, specifically in the context of communication and its evolutionary necessity. If this is the case, then it seems imperative that current FPSs portray it accurately; we know of no current FPSs that portray the four natural intervals identified here. The current study identifies a unique and shared progression in extreme facial pain expression in infants that current metrics do not reflect. Future research should address the significance of these natural intervals on pain metrics. For example, do these shared patterns mark or coincide with self-reported pain intensity in older children? Are there possible thresholds in the pain experience associated with corresponding expression or communicative patterns? Does a more accurate portrayal of the expression in an FPS affect the validity of its scores? That is, would improving the faithfulness of the pain display aid in the communication of pain with younger children who are cognitively challenged by current tools? The small number of cases studied here limits application of the findings. Additionally, there may be other temporal subtleties at the point-pair level or movement in other areas not captured by this “upper versus lower face” approach. However, these findings call attention to a need to take a more objective approach to the future development and refinement of pain measurement tools such as FPSs.

## 5. Conclusions

There appears to be both shared morphology and temporal dynamics of movement in the occurrence of the PFP across the first expressive cycle in newborns expressing more extreme facial intensity. These commonalities in the pain display further support the universality of the pain expression, and may help to identify patterns, and thus inform the objective development and improvement of faces pain scales in terms of more anatomically faithful intervals. A more accurate graphic portrayal in FPSs, true to natural patterns, may facilitate the communication and measurement of pain in younger children.

## Figures and Tables

**Table 1:** Commonly published pediatric clinical pain tools employing facial expression [3, 7, 16].

Behavioral, Observational, or Physiological Pain Scales	
BIPP	Behavioral Indicators of Infant Pain
CHEOPS	Children's Hospital of Eastern Ontario Pain Score
CHIPPS	Children's and Infant's Postoperative Pain Scale
CRIES	Crying, Requires oxygen administration, Increased vital signs, Expression, Sleeplessness
EDIN	Échelle Douleur Inconfort Nouveau-Né (neonatal pain and discomfort scale)
FLACC	Face, Legs, Activity, Cry, and Consolability
NIPS	Neonatal Infant Pain Scale
NPASS	Neonatal Pain Agitation and Sedation Scale
PIPP	Premature Infant Pain Profile

*Faces Pain Scales *	

FACES	Wong-Baker FACES Pain Rating Scale. Cartoon “happy” to “sad” depictions
FPS-R	Faces Pain Scale—Revised. Symbolic line drawing representations
OUCHER	Oucher Scale. Photographic depictions, 3 ethnic versions

**Table 2:** Characteristics of the 3 infants (selected from 63) showing the greatest intensity of facial expression.

Infants with most facial movement	Display intensity (change from baseline to peak % facial width)*	Duration of expression (time in seconds from baseline to peak)	Time points analyzed (number of frames at 11.6 frames per second)	Demographics (sex, age, gestational age)
Infant A	80.69	3.88	45	Male, 40.5 hours, 41 weeks
Infant B	55.46	3.97	46	Female, 37.5 hours, 39 weeks
Infant C	65.24	1.81	21	Male, 49.0 hours, 40 weeks

*Total sample (*n* = 63) minimum 4.49, maximum 80.69, mean 30.89, standard deviation 15.35.
